# A novel patient-Centered approach to clinical trial readiness in rare diseases: Application in Aicardi-Goutières Syndrome (AGS)

**DOI:** 10.1016/j.ymgme.2026.109765

**Published:** 2026-02-08

**Authors:** Anjana Sevagamoorthy, Francesco Gavazzi, Zarrin Tashnim, Peter Hong, Ylenia Vaia, Min Ae Lee-Kirsch, Despina Eleftheriou, Shanice Beerepoot, Marie Hully, Elizabeth M. Berry Kravis, Pamela Ventola, Melissa Raspa, Anne Wheeler, Sara B. DeMauro, Allan M. Glanzman, Elise Townsend, Tina Duong, Stacy Cusack, Ann T. Harrington, Samuel Pierce, Michelle Fitzgerald, Elisa Fazzi, Jessica Galli, Simona Orcesi, Davide Tonduti, Evangeline Wassmer, Devon Cordova, Laura A. Adang, Cherie Butts, Adeline Vanderver

**Affiliations:** a Neurology Department, Children's Hospital of Philadelphia, Philadelphia, PA, USA; b Department of Pediatrics, University of Pennsylvania, Philadelphia, Philadelphia, PA, USA; c Neuroscience research center, Department of Biomedical and Clinical Sciences, University of Milan, Milan, Italy; d Department of Pediatrics, University Hospital and Medical Faculty Carl Gustav Carus, Dresden University of Technology, Dresden, Germany; e University Center for Rare Diseases, University Hospital and Medical Faculty Carl Gustav Carus, Dresden University of Technology, Dresden, Germany; f German Center for Child and Adolescent Health (DZKJ), partner site Leipzig/Dresden, Germany; g Department of Infection, Immunity & Inflammation, University College London and Great Ormond Street Hospital London, UK; h Division of Child Neurology, VUmc, Amsterdam, Netherlands; i Department of Pediatric Neurology, Necker Hospital, APHP Centre Paris, France; j Department of Pediatrics, Rush University, IL, USA; k Child Study Center, Yale School of Medicine, CT, USA; l RTI International, NC, USA; m Division of Neonatology, Children's Hospital of Philadelphia, Philadelphia, PA, USA; n Department of Physical Therapy, Children's Hospital of Philadelphia, Philadelphia, PA, USA; o MGH Institute of Health Professions, Boston, MA, USA; p Stanford Medicine, CA, USA; q Children's Hospital of Philadelphia, Philadelphia, PA, USA; r Department of Clinical and Experimental Sciences, University of Brescia, Italy; s Child Neurology and Psychiatry Unit, ASST Spedali Civili of Brescia, Italy; t Department of Brain and Behavioral Sciences, University of Pavia, Italy; u Child Neurology and Psychiatry Unit, IRCCS Mondino Foundation, Pavia, Italy; v Unit of Pediatric Neurology, C.O.A.L.A (Center for Diagnosis and Treatment of Leukodystrophies), V. Buzzi Children's Hospital, Milan, Italy; w Department of Biomedical and Clinical Sciences, Università degli Studi di Milano, Italy; x Neurology Department, Birmingham Children's Hospital, Institute of Health and Neurodevelopment, Aston University, Birmingham, UK; y Aicardi-Goutières Syndrome Advocacy Association (ASGAA), USA; z Department of Neurology, Perelman School of Medicine, University of Pennsylvania, Philadelphia, PA, USA; aa Biogen, Cambridge, MA, USA

**Keywords:** Clinical trial readiness, Rare disease drug development, Patient-focused drug development, Clinical outcome assessments, Aicardi Goutières syndrome, Concepts of interest

## Abstract

**Introduction:**

Aicardi-Goutières Syndrome (AGS) is a genetic type 1 interferonopathy that causes white matter abnormalities and intracranial calcifications, resulting in varying degrees of neurologic impairment and systemic manifestations. Novel disease-modifying therapies for AGS are forthcoming. The 2022 Food and Drug Administration guidance, “*Patient-Focused Drug Development*” (PFDD), emphasizes the importance of including patients' voices early in the design of clinical trials. This represents an urgent unmet need in rare disease research. In this study, we propose and pilot a new methodology to identify patient-centered Concepts of Interest (COIs) and suitable Clinical Outcome Assessments (COAs) for clinical trials.

**Methods:**

The study was performed under the Myelin Disease Biorepository Project within the Global Leukodystrophy Initiative Clinical Trial Network. A sequential multicomponent approach, piloted in AGS, was designed to (i) identify COIs, (ii) select COAs capable of measuring the COIs through expert consensus, and (iii) assess the feasibility of COA application. Experts were identified based on relevant scientific publications and expertise in AGS (disease experts for COI) and/or their application of relevant COAs (outcome experts for COA). Expert consensus was achieved using the modified eDelphi approach for COIs, expertise-specific multi-panel focus group discussions, and pre-and post-surveys for COA selection. Consensus was defined as ≥70% agreement among the experts. This was followed by a virtual stakeholder discussion with patients and/or patient representatives to assess the feasibility of the COA application in the context of a clinical trial.

**Results:**

Based on the health priorities identified by patient caregivers, the proposed approach revealed a set of fit-for-purpose COIs across the motor, adaptive behavior, and neurologic functional domains. All experts acknowledged the significance of each caregiver-identified priority but expressed differing opinions on the likelihood of observing changes in the functional domain within a 6- to 12-month timeframe. Following this, a consensus-building approach for COA selection for each identified COI resulted in a paired COI-COA panel applicable to future AGS clinical trials. Finally, the discussion on the feasibility of application of the selected COAs with the patients and/or patient representatives elicited critical information to design a patient-centered prospective COA protocol, applicable to clinical trials and natural history studies.

**Discussion:**

The proposed approach marks the first step toward a patient-centered clinical trial design for rare diseases. It establishes a paired COI-COA panel, as well as informs the design of a patient-centered prospective COA protocol for upcoming AGS clinical trials and natural history studies. Additionally, the identified COA panel facilitates the creation of a multicomponent endpoint for clinical trials, which is especially crucial in phenotypically diverse disorders like AGS. This approach is widely applicable across leukodystrophies and rare diseases.

## 1. Introduction

Aicardi-Goutières Syndrome (AGS) is a type 1 interferonopathy of genetic origin. [1,2] To date, pathogenic variants in nine genes have been associated with AGS: *TREX1*, *RNASEH2B*, *RNASEH2C*, *RNASEH2A*, *SAMHD1*, *ADAR*, *IFIH1*, *LSM11*, and *RNU7*–*1.* [3-9] Affected individuals typically present with early-onset encephalopathy, microcephaly, intracranial calcifications, and white matter abnormalities with varying degrees of neurologic impairments [10-13] accompanied by heterogeneous systemic involvement. [10,11,13] With the advent of next-generation sequencing, late-onset (> 12 months) sub-forms have also been identified and described in the literature. [10,14-18]

A growing understanding of AGS pathogenesis has led to repurposing existing drug candidates, such as baricitinib, other Janus Kinase In-hibitors (JAKi), and interferon receptor blockade (anifrolumab). [19-28] In addition, ongoing innovations suggest the potential for other novel disease-modifying drug candidates for this rare disorder. However, clinical trials in rare diseases, such as AGS, entail numerous challenges, including the genotypic and phenotypic heterogeneity of the diseases, which necessitate investigation by sub-cohorts and access to fewer subjects for enrollment, resulting in small-sized cohorts for trials. [29] Furthermore, avoiding floor effects in clinical outcome measures may be difficult in rare diseases, while the measurement of skills using norm-referenced tests may be insensitive to small changes in performance. [30] Selecting appropriate clinical outcome assessment tools and determining clinically relevant endpoints are particularly important for successful drug-candidate clinical trials in this rare disease context. [29,31,32]

In 2022 and 2023, the Food and Drug Administration (FDA) published a four-part guidance series on Patient-Focused Drug Development (PFDD). [33] This series emphasizes the significance of a patient-centered evaluation of clinical trial outcomes. In this guidance, the FDA emphasizes the importance of incorporating the patient's voice early in clinical trial design to ensure the selection of clinical outcomes that are meaningful to patients. This process requires a formal approach to identifying the health priorities of patients and caregivers (e.g., lower limb function) and determining relevant disease-related concepts of interest (COI) (e.g., the ability to walk) that are both meaningful and changeable within the context of a clinical trial, as well as selecting clinical outcome assessments (COA) that effectively capture the COI. Considering the recent FDA guidance, embracing a patient-centric approach to clinical trial readiness is an urgent and unmet need in rare disease research.

In this study, we aim to address this gap in the context of AGS. Using qualitative and quantitative approaches, our team identified health priorities derived from AGS patient caregivers as a first step. [34] Building on this prior study, we propose and pilot a methodology for identifying COIs and selecting COAs tailored to AGS in alignment with the FDA's Patient-Focused Drug Development framework. [33] This study will advance clinical trial readiness for AGS by establishing a panel of COAs applicable to AGS clinical trials and supporting COA protocol development that addresses the unique challenges of rare disease trials while prioritizing meaningful, patient-centered outcomes.

## 2. Materials and methods

**Fig. 1.**
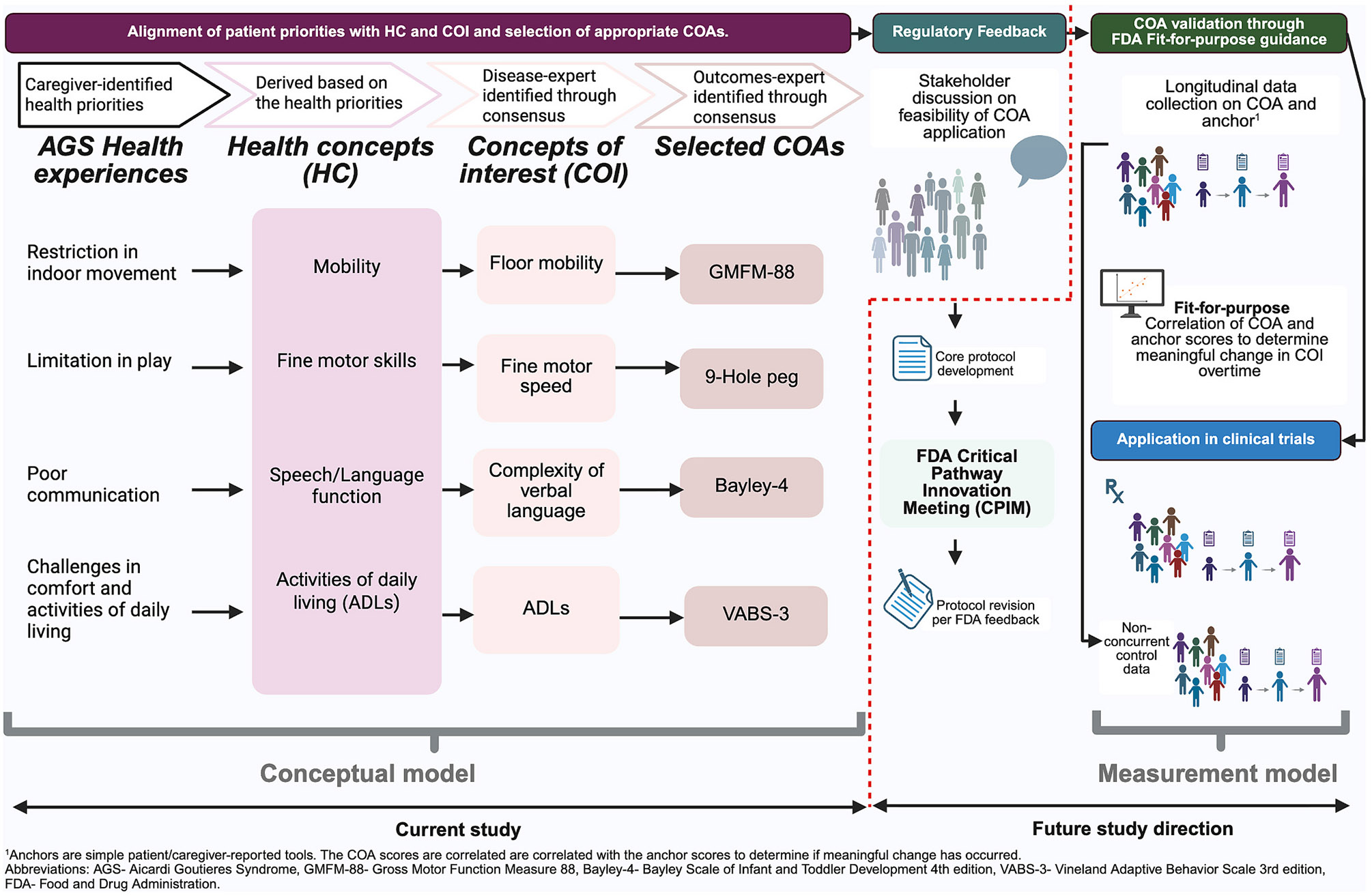
Overall framework of the novel patient-centered approach to clinical trial readiness Created in https://BioRender.com

The study was performed under the Children's Hospital of Philadelphia (CHOP) Institutional Review Board (IRB) approved Myelin Disease Biorepository Project (MDBP) (IRB 14–011236). A sequential multicomponent approach was designed in the form of a workshop and included (i) COI identification followed by (ii) COA selection. Each component consisted of an initial virtual group discussion, followed by expert consensus building through a modified eDelphi methodology [35] for COI identification, and blinded pre- and post-surveys accompanying focus group discussions for COA selection. Finally, a stakeholder discussion with patients and/or patient representatives was performed to assess the feasibility of the completion of the selected COAs and solicit input on considerations when designing a core prospective study protocol. The overall framework of the proposed sequential multicomponent approach is illustrated in Fig. 1.

### 2.1. Identification of experts

Experts were identified based on their contributions to the field through published work and experience caring for AGS-affected individuals (for disease experts) and/or expertise in applying relevant clinical outcomes (for outcome experts). The identified experts were also invited to suggest other experts in the field to serve on the panel. Adequate representation by COA-based expertise (physician, neuropsychologist, and therapist) was considered during the outcome expert selection process. A study team member compiled the list of experts and contacted each eligible individual to solicit their participation in the workshop. Upon acceptance of the invitation to participate in the study, video consents were obtained from each expert to record the virtual group discussions.

### 2.2. Identification of health concepts (HCs)

HCs were defined based on health priorities identified by patient caregivers in the context of a prior study that explored the impact of AGS on affected individuals and their families. [34] In this study, health priorities were elicited using a multimethod approach that employed Section 7 of the Caregiver Priorities and Child Health Index of Life with Disabilities (CPCHILD), [36] as well as qualitative patient-caregiver interviews. The CPCHILD is an Observer Reported Outcome ObsRO and includes six domains: Personal Care / Activities of Daily Living, Positioning, Transferring and Mobility, Comfort and Emotions, Communication and Social Interaction, Health, and Overall Quality of Life. Under Section 7 of CPCHILD (Importance of Items to Quality of Life), caregivers rank items within each domain on a Likert scale ranging from 0 (Least Important) to 5 (Most Important), informing the determination of patient health-related priorities for change and improvement.

Conjoint analysis of Section 7 of CPCHILD showed representation of all CPCHILD domains within the top 10 priorities identified by the caregivers, with a relatively higher priority to items related to communication, overall health, and comfort. [34] In addition, analysis of the qualitative data elicited caregivers' hopes and priorities for improvement in functional abilities of the affected child and overall happiness, as well as independence for a better quality of life. [34] This included hopes for improvements in motor skills, activities of daily living, and happiness and comfort. [34] Based on these findings, we defined HCs at the domain level as gross and fine motor abilities, cognitive and psychomotor abilities, and adaptive behavior abilities, which included communication, activities of daily living, and social skills. The caregiver-identified health priorities and HCs were then presented to the panel of disease experts in the context of a virtual focus group discussion to identify specific COIs relevant to each HC:

### 2.3. Identification of COI from HCs

This component consisted of two steps: (Step 1) the exploration and compilation of all clinical domains of interest applicable to clinical trials through a one-time virtual group discussion, and (Step 2) consensus building among experts using the modified Delphi approach. The modified Delphi methodology is a systematic and structured approach to building consensus among experts using predefined topics for discussion. [35] It is an iterative process involving multiple rounds of voting on pre-specified themes by an expert panel. [35] Modified eDelphi is a real-time Delphi process hosted and facilitated virtually through online software. [37]

#### 2.3.1. COI focus group discussion

A total of 6 disease experts participated in the initial virtual group discussions. The focus group discussion was video recorded, during which the facilitator (F.G.) introduced the overall objectives of the workshop, including the goal of the focus group session, and outlined the expectations of the eDelphi process to the group of experts. Following this, the facilitator presented the caregiver-identified health priorities elicited in the prior study [34] and the HCs to the panel for discussion, and solicited responses on the clinical outcomes of interest within each domain as they relate to clinical trials. In addition, disease experts were invited to suggest domains that they perceived as relevant, leading to the inclusion of “Neurologic signs” as an additional domain. As the experts suggested critical clinical outcomes for AGS within each of the suggested domains, the facilitator asked clarifying questions to confirm responses and definitions, as applicable.

#### 2.3.2. Generation of statements

Upon completion of the expert focus group discussions, the facilitator generated statements for each clinical outcome that emerged from the discussion based on the following parameters: (a) *importance and changeability* of COIs and (b) *measurability* of COAs in the context of clinical trials. Importance was defined as the relevance of the clinical outcome to the affected individual from a quality of life and clinical trial perspective. For example, “Independent ambulation is important to the patient.” Changeability indicates the likelihood of a change in the clinical outcome with a potential disease-modifying intervention while accounting for the time and feasibility of the change. The duration was 12 months from the initiation of the intervention. E.g., “Independent ambulation can improve in 12 months with the intervention”. Measurability was defined as the COA's ability to accurately measure the COI in a clinical trial setting.

#### 2.3.3. Modified E-Delphi process

The modified eDelphi approach was employed to identify COI and hosted virtually on the eDelphi.org software^®^. [38] This tool facilitates blinded and structured communication to build consensus while providing the infrastructure to organize the communications. Before programming the generated statements, the facilitator completed the required training to use the software, grouped by the themes, into the project platform. In addition, a comment box was included under each theme to enable capturing of additional information. The project was set up to anonymize the responses, both for the facilitator and the participants, to ensure blinding to avoid potential influence or coercion when voting on the statements. The experts were added to the project using their electronic mailing addresses, and the initial round of invitations was sent out to cast their votes on the eDelphi platform.

**Fig. 2.**
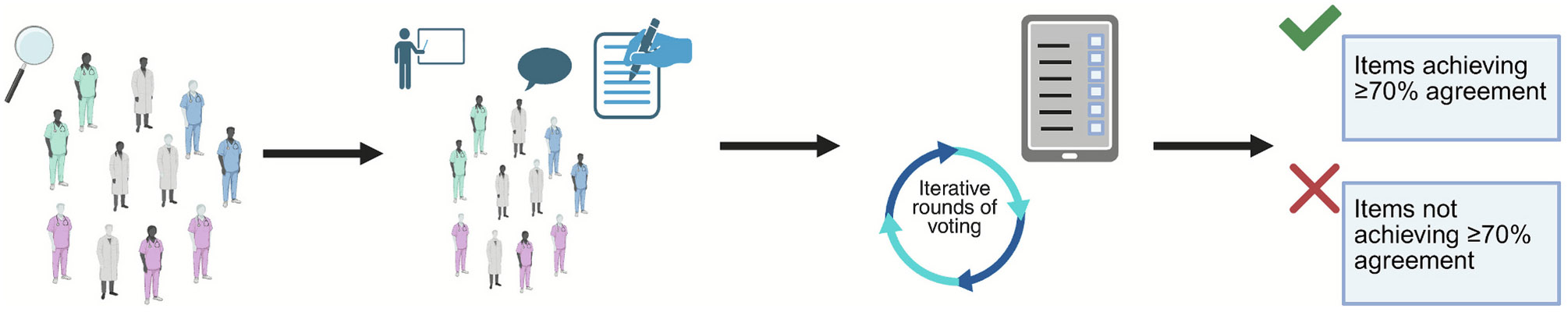
Consensus-building approach using the eDelphi methodology. Created in https://BioRender.com

The entire process was performed over three months and included four rounds, each lasting an average of three weeks. Up to 12 of 13 disease experts participated in each round of the eDelphi process (round 1: *N* = 10; round 2: *N* = 12; round 3: N = 10; round 4: N = 12). The experts were asked to vote on the generated statements in each round. In case of disagreements with any statements, the participants were required to include comments providing the rationale for their dispute. At the end of each of the first three rounds, the facilitator exported the data from the voting process and analyzed the output to determine the qualification of the clinical outcomes. A clinical outcome was considered qualified upon reaching a 70% consensus on its importance and changeability within 12 months. The outcomes that qualified were not subject to subsequent rounds of voting. When consensus was not achieved, the facilitator rephrased or modified the statements based on the participant's comments before deploying the outcomes for the next round of voting. At the end of the fourth round of voting, the final output of the voting process was consolidated, and the clinical outcomes that did not achieve a 70% consensus were excluded from further study (Fig. 2).

### 2.4. Selection of COAs for COIs

The COA selection component followed the identification of COIs and included an initial virtual group discussion (Step 1) among outcome experts to generate a list of COAs capable of measuring the identified COIs. This was then followed by a series of expertise-based focus group discussions (Step 2) conducted between pre- and post-surveys to select COAs.

#### 2.4.1. Initial virtual group discussion

A total of 10 outcome experts participated in the initial COA group discussions. A study team member (F.G.) organized and facilitated this discussion session. Like the COI focus group, the facilitator introduced the workshop's goal, including the group discussion, and described the expectations for the subsequent consensus-building process to the outcome experts. Following this, the facilitator presented each COI that passed consensus to the panel for discussion and compiled a list of all suggested COAs capable of measuring each identified COI. At the end of both group discussions, the facilitator consolidated and presented the themes to the participants for any final suggestions before moving forward with the consensus-building process.

#### 2.4.2. Consensus building for COA selection

Upon compilation of all expert-identified COAs, the facilitator (FG) grouped the COAs based on the tool-specific expertise required for their application. Three tool-specific subpanels were then formed: physician, neuropsychologist, and therapist (both physical and occupational therapists). Sixteen outcome experts, five neurologists, five neuropsychologists, and six therapists (with three from each occupational and physical therapy specialty) participated in the consensus-building approach to select COAs. Focus group discussions were facilitated for each subpanel and sandwiched between subpanel-specific anonymized surveys programmed on PollEverywhere.com^®^ [39]. In both surveys, a 3-point Likert scale format for the response options was opted for and included “Agree,” “Neutral,” and “Disagree.” The pre-focus group survey consisted of statements generated on the ability of each COA to measure the COI of interest in a clinical trial. Experts in each subpanel were invited to complete the subpanel-specific survey before the focus group discussion. The responses logged on to the pre-survey were then presented to the sub-panelists, who were asked to comment on the responses and suggest alternative tools, if applicable. The feedback was then integrated into the post-focus group survey and administered to the expert subpanel. Consensus was considered to have been achieved for COAs in the context of a COI upon reaching an agreement of ≥70% in the post-focus group survey.

### 2.5. Stakeholder discussion on feasibility of COA application

Upon identification of the AGS-specific COI-COA panel, we organized a virtual group discussion with patients and patient representatives. The group discussion explored the feasibility of application of the selected COAs from the patient's perspective, as well as perceived logistical barriers/facilitators to participation in a COA from the context of a clinical trial. Patients and caregivers of those enrolled in the MDBP protocol were invited to the group discussion, yielding a total of 5 participating families. The group discussion was led by a member of the study team (AS & FG) who facilitated the discussion. The facilitator initially presented the overall goal of the group discussion, the recent FDA guideline, outlined the identified COI-COA panel, as well as the key information pertinent to the COAs, to the participants. Following this, the facilitator solicited feedback on perceived COA feasibility and barriers to participation in the selected COAs from the context of clinical trials, and in addition, probed for insight into potential measures to alleviate the perceived barriers to participation. At the end of the discussion, the facilitator consolidated and summarized the outcomes of the discussion to the participants, verified appropriateness, and solicited additional feedback prior to concluding the session.

### 2.6. Statistical analysis

Proportions were computed to determine consensus. Consensus was achieved upon reaching an agreement of ≥70% among the experts.

## 3. Results

### 3.1. Consensus building to identify COI and select COAs

**Fig. 3.**
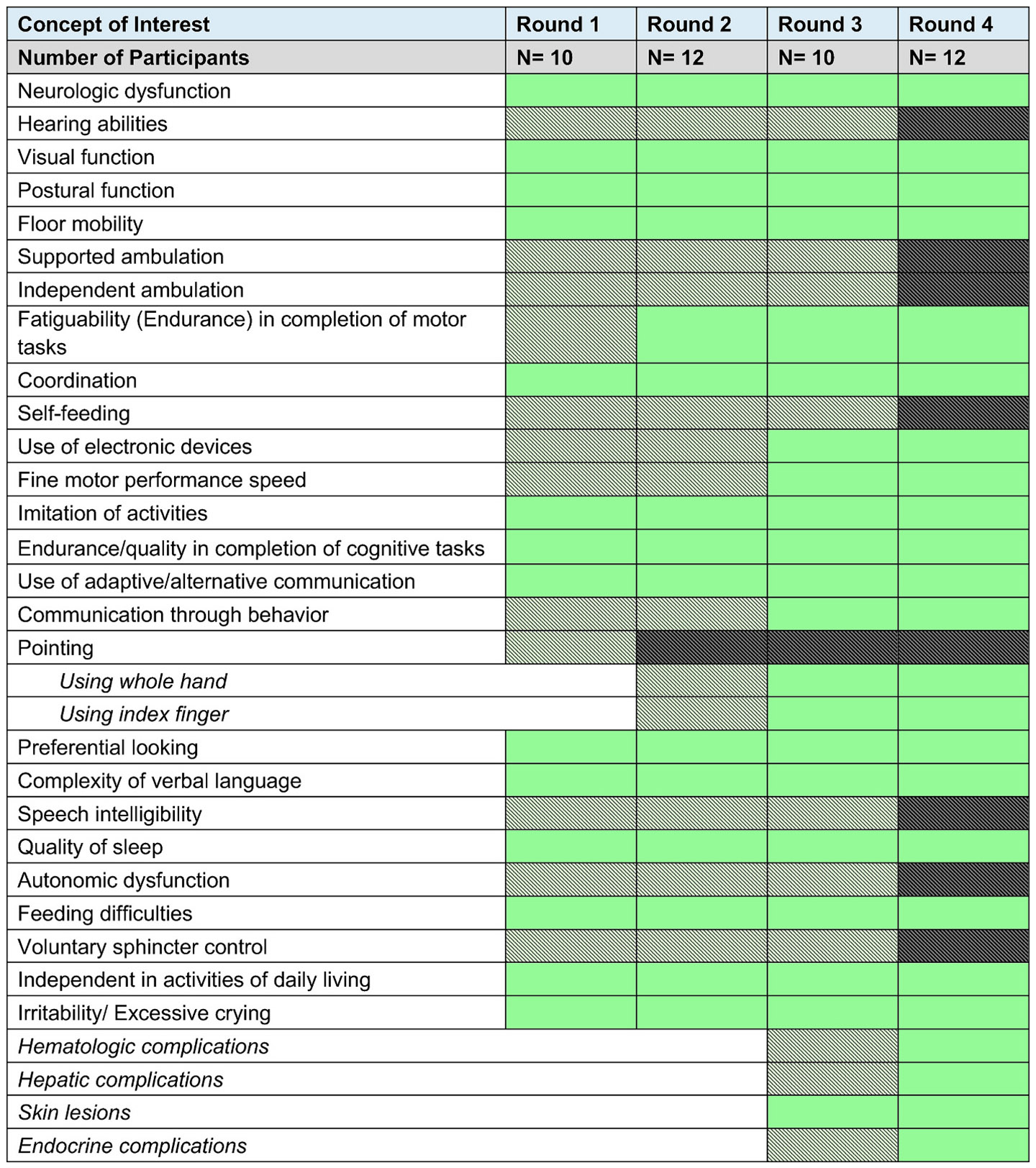
Concept of Interest (COI) identification Using Modified eDelphi Approach Green indicates that the domain has passed consensus, Shaded grey indicates that the domain was being voted upon, and Shaded black indicates the domain failed consensus.

The process of consensus building among disease experts to identify the COIs is detailed in Fig. 3, and the chosen COAs are summarized in Table 1 alongside the corresponding COI. In addition, all suggested COAs and those achieving consensus on the multi-panel focus group discussions among outcome experts are presented in **Supplemental files 1–3.**

#### 3.1.1. Motor function

In total, the disease experts identified 10 domains of motor function during the focus group discussion. Of these, four outcomes were purely gross or fine motor skills, and two required both gross and fine motor skills. The four gross motor outcomes identified were postural functions (head and trunk control), floor mobility (achieved by either rolling, scooting, or crawling to a target), supported (achieved by either cruising or with the use of a walker), and independent (regardless of the quality of ambulation) ambulation. The fine motor outcomes identified were self-feeding, use of electronic devices, fine motor performance speed, and pointing. Additionally, clinical outcomes requiring both gross and fine motor skills identified were endurance (fatiguability) in completing motor tasks and coordination during play.

Postural function, floor mobility, and coordination in play achieved consensus at the end of the first round of voting. In addition, per the disease expert's suggestion at the end of round 1, pointing was split into two domains— using the whole hand and index finger at the end of round 1. Following this, the consensus was reached for endurance (fatiguability) in completing motor tasks in round 2, the use of electronic devices, fine motor performance speed, and pointing with the whole hand and index finger in round 3. At the end of round 4, self-feeding, supported ambulation, and independent ambulation did not achieve consensus due to a lack of agreement on changeability and were therefore excluded. Quoting an expert on changeability regarding supported and independent ambulation, “*the possibility of improvement within 6 to 12 months depends on the age of the child and the stage of the disease.*”

Among the gross motor COIs identified, consensus on COA selection was achieved only for postural function and floor mobility. In clinical trials, the Hammersmith Infant Neurologic Examination (HINE) [40,41], Bayley Scales of Infant and Toddler Development- 4th edition (Bayley-4), [42,43] and Gross Motor Function-88 (GMFM-88) scale [44] were identified as the best measures of both postural function and floor mobility. In addition, the physician panelists identified the AGS severity scale [45] and wearables as measures of postural function and floor mobility in clinical trials, respectively.

#### 3.1.2. Adaptive behavior (including communication)

Disease experts identified eight domains of interest, which included communication through behavior, use of adaptive/alternative communication, complexity of verbal language, speech intelligibility, endurance and quality in completion of cognitive tasks, independence in completion of activities of daily living, imitation of activities, and preferential looking. At the end of round 1, the following domains reached a consensus— the use of adaptive/alternative communication, the complexity of verbal language, endurance, quality in completing cognitive tasks, independence in completing activities of daily living, imitation of activities, and preferential looking. Communication through behavior achieved consensus in round 3. Speech intelligibility did not attain consensus due to a lack of agreement on changeability during the eDelphi process. An expert stated, “*It's rare to observe an improvement of this speech component.*”

The outcomes experts collectively agreed on COAs for all identified COAs in this domain, except for using adaptive/alternative communication, endurance, and quality in completing cognitive tasks. The Denver Developmental Scale- 2nd edition, [46] the Bayley Scales of Infant and Toddler Development – 4th edition (Bayley-4), and the Vineland Adaptive Behavior Scale- 3rd edition (VABS-3) [47] were identified as the best measures of complexity of verbal language. In addition, the VABS-3 was also selected as the best tool to measure communication through behavior, imitation of activities, and independence in completing activities of daily living. Another measure of independence in completing activities of daily living was the Pediatric Evaluation of Disability Inventory Computer Adaptive Test (PEDICAT) [48] identified by the therapists. The neuropsychology panelists collectively agreed on the Bayley-4 as a measure of the complexity of verbal language. The eye tracker using the preferential looking protocols achieved consensus among physician panelists as a measure of preferential looking.

#### 3.1.3. Neurologic function

The disease experts identified five domains of neurologic function: hearing and visual function, neurologic dysfunction such as muscle tone and movement abnormalities, autonomic dysfunction, and voluntary sphincter control. The consensus was achieved only for visual function and neurologic dysfunction during the eDelphi process, which occurred at round 1. Hearing function, autonomic dysfunction, and voluntary sphincter control failed to reach an expert consensus on changeability. In the context of the changeability of autonomic dysfunction and voluntary sphincter control, experts expressed concern about the possibility of change within 6 to 12 months.

Of the two identified COIs, COAs were selected only for neurologic dysfunction and included the AGS Severity Scale and the HINE. The outcome experts suggested the Neonatal Assessment of Visual European Grid (NAVEG) [49] as a potential measure of visual function, but this failed to achieve consensus among the experts.

#### 3.1.4. Systemic complications

Two domains were identified during the focus group discussion: sleep quality and irritability/ excessive crying. Both achieved consensus at the end of round 1. During the eDelphi process, the experts suggested including the following systemic complications of AGS: hematologic, hepatic, endocrine, and skin. The experts voted on this in rounds 3 and 4, with skin lesions achieving consensus in round 3 and the remaining (hematologic, hepatic, endocrine) in round 4.

Existing standardized tests measure sleep quality and hematologic, hepatic, endocrine, and skin lesions, but they were not presented to the outcome experts for discussion. Irritability/ excessive crying did not elicit a COA among the initial focus group discussion experts.

### 3.2. Stakeholder (patient and patient caregivers) input

The participants indicated familiarity with most of the selected COAs from either direct participation or, as applicable, based on their child's participation in the COA in the past. Participants did not express concerns related to the feasibility of completing the selected COAs. On the other hand, insisted on the inclusion of as many standardized assessments as possible, while eliminating redundancy between assessments. They also suggested tandem or concurrent application of different COAs by combining physical and occupational therapy assessments. A few participants suggested customizing the test to the children's abilities, such as the use of a Rifton chair for appropriate posturing of the child when assessing function using an eye gaze device. Several families also suggested incorporating the input of their local therapy teams as a post-hoc reference. Families also suggested several considerations when designing a prospective protocol, such as scheduling of endurance assessments earlier in the day, avoiding assessment of performance outcomes (PerfO) after any invasive procedure, and accommodating just-enough breaks between assessments to allow the subjects to recoup. Finally, families expressed concerns with travel and lodging expenses incurred to accommodate the assessments and cited remote assessments to be invaluable. From the perspective of a prospective natural history study, the dissemination of short videos illustrating the COA performance prior to the scheduled administration was recommended.

## 4. Discussion

The approach detailed in this study is novel and closely aligns with the current FDA guidelines on patient-centric clinical trial design. Identifying patient caregiver priorities early in drug development for AGS allows for the integration of patient input in the determination of clinical trial outcomes that are important to affected individuals. In addition, this methodology permits input from disease experts on its application in clinical trials, thereby establishing the Context-of-Use (COU). Thus, this coupled approach, incorporating both patient/care-giver and disease expert input, is likely to yield COIs that are most important to patients/caregivers and adequately specific and responsive enough for use in clinical trials. In addition, LDs are a group of largely heterogeneous disorders that impact multiple domains of neurologic function to varying degrees and are not always predictable using genotype alone. Hence, a multicomponent endpoint capable of measuring multiple COIs is ideal. The approach to COA selection used in this study yields a disease-specific COA panel that enables optimal endpoint design for use in clinical trials and can be used a model for identifying COIs for trials with other rare diseases.

During COI identification, all disease experts unanimously agreed on the “importance” of each caregiver priority-derived clinical domain. Any failure to achieve consensus on these domains was primarily driven by a lack of agreement on “changeability within 6 to 12 months”, a standard timeline applied in randomized controlled clinical trials to measure efficacy. Consensus on COIs was achieved predominantly in the language, systemic disease, and fine motor domains. This aligns closely with the existing literature surrounding treatment with Janus Kinase Inhibitor (JAKi) in AGS, which suggests improvements in systemic disease, social, language, and fine motor domains, with sustained gross motor deficits. [21-24] Mainly, supported and independent ambulation did not pass consensus, with experts noting concerns around changeability in ambulation within the defined timeframe and in the context of disease severity. This has been reported in the literature, which shows that AGS disease severity and developmental skills attained at the initiation of treatment and genotype play a key role in treatment efficacy. [22,24]

Most COAs identified by experts to the selected AGS panel have been previously applied to leukodystrophy clinical trials (Table 1), which is encouraging in its potential for applicability in AGS clinical trials. The COA selection process yielded predominantly Performance Outcomes to the AGS COA panel. The Observer/Patient Reported Outcomes (ObsRO/PROs) identified were VABS-3 and PEDICAT. VABS-3 achieved unanimous consensus, particularly in the communication and activities of daily living domains. Despite evidence of higher cognitive function than previously perceived in otherwise neurologically impaired children and a high correlation with VABS-3 as a measure of non-verbal communication, [52] Leiter-3 did not pass expert consensus, which may be explained by its limited applicability in children younger than 3 years of age, and the degree of administration failure in case of severe neuro-cognitive impairment. Similar concerns around applicability across ages and spectrum of disease severity with potential for significant floor effect on COAs in more severely affected AGS individuals were also noted in the context of other COAs that failed consensus. This highlights the underlying challenge associated with clinical trial design in rare diseases such as AGS and evaluation of treatment efficacy [10], as well as the potential need to design more tailored COA to capture functional abilities within this specific population. Nonetheless, the COA panel identified using this approach enabled the development of a multicomponent endpoint applicable in evaluating treatment efficacy in a phenotypically heterogeneous cohort.

This approach has limitations. First, ascertainment bias among disease experts from variable exposures to patients across disease severity is possible, at least partially determined by the local patient population and clinical practices in case identification. To limit this concern, we intentionally recruited experts from institutions across multiple geographic locations. Second, the consensus-building approach for COA selection necessitates all experts in the panel or subpanel, as in this study, to be knowledgeable regarding the scale under discussion. However, this may not always be the case, impacting the expert's ability to provide input on the scale. To circumvent this, each expert in the subpanel was provided detailed information on each COA to reference during participation. Despite this, a lack of experience in applying the tool itself remains and could potentially impact the results of the voting process.

This is the first paper to outline a methodological approach, basing the recent FDA guidelines on “Patient-Focused Drug Development”, to incorporate patient/patient caregiver inputs on health priorities and COA application for clinical trial design. The approach is broadly applicable across leukodystrophies and rare diseases. Notably, clinical trial readiness for heterogeneous diseases yields a panel of COAs to develop a multicomponent endpoint that measures a broad spectrum of COIs, and informs the design of a patient-centered prospective COA protocol for application in a standardized manner across both clinical trials and natural history studies. As a next step, we plan to develop a core prospective COA protocol and solicit FDA's feedback in the context of a Critical Path Innovation Meeting (CPIM). This approach is scalable across rare diseases, including leukodystrophies and will enable clinical trial readiness for rare and ultrarare diseases with patient centricity at its core.

## Supplementary Material

2

3

4

## Figures and Tables

**Table 1:** Summary of selected Clinical Outcome Assessments (COAs) and Concepts of Interest (COIs).

COIs	COAs	Validated for age range (years)	Validated for leukodystrophies	Previously applied in leukodystrophies (*prior application in CT trials)	Applied in AGS	COA type
**Physician Panel (*N* = 5)**						
Postural Function (head and trunk) Neurologic dysfunction	AGS Severity Scale [45]	All	Yes	Yes (*NCT04517253)	Yes [45]	ClinRO
Complexity of verbal language	Denver Developmental Scale -2nd edition [46]	0–6	No	Yes (*NCT01838941)	Yes [10]	Other (Developmental Screening tool)
Preferential looking	Eye tracker (Preferential Looking Protocols) [50]	NA	No	No	No	PerfO
Postural Function Floor mobility Neurologic dysfunction	Hammersmith Infant Neurologic Examination (HINE) [40,41]	0.17–2	No	Yes (*NCT02396459)	Yes	PerfO
Floor mobility	Wearables	NA	No	No	No	PerfO
**Neuropsychologist panel (N = 5)**						
Complexity of verbal language Postural Function (head and trunk) Floor mobility	Bayley Scales of Infant and Toddler Development- 4th edition [42]	0–3.5	No	Yes (*NCT04771416)	No	PerfO
Fine motor speed	Developmental Neuropsychological Assessment- 2nd edition (NEPSY-II) [51]	3–16.93	No	Yes	No	PerfO
Communication through behavior Complexity of verbal language Imitation of activities Independence in completion of activities of daily living	Vineland Adaptive Behavior Scale- 3rd edition [4^7^]	0–90	Yes	Yes (*NCT04283227)	Yes [52]	ObsRO/PRO
**Therapist panel (*N* = 6)**						
Fine motor speed	9-Hole peg [53]	3–85	No	Yes (*NCT04849741)	Yes	PerfO
Independence in completion of activities of daily living	Pediatric Evaluation of Disability Inventory Computer Adaptive Test (PEDICAT) [48]	0–20	No	Yes	Yes	ObsRO/PRO
Fatiguability (Endurance) in completion of motor tasks	6 Minute Walk Test (6MWT) [54]	2–65+	No	Yes (*NCT03124459)	Yes	PerfO
Floor mobility Postural Function (head and trunk)	Gross Motor Function Measure-88 (GMFM-88) [44]	0.43–16	Yes	Yes (*NCT01303146)	Yes [55]	PerfO

Abbreviations: COAs (Clinical Outcome Assessments); COIs (Concepts of Interest); ClinRo (Clinician Reported Outcome); PerfO (Performance Outcomes); ObsRO/PRO (Observer Reported/Parent Reported Outcome).

* Prior use in LD trials identified through review of ClinicalTrial.gov and European Union Drug Regulating Authorities Clinical Trial Network.

## Data Availability

No data was used for the research described in the article.

## Appendix A. Supplementary data

Supplementary data to this article can be found online at https://doi.org/10.1016/j.ymgme.2026.109765.
