# Supplementary material for: A novel patient-Centered approach to clinical trial readiness in rare diseases: Application in Aicardi-Goutières Syndrome (AGS)
Source: Mol Genet Metab. Author manuscript; Available in PMC 2026 May 12. (PMC13162174; doi:10.1016/j.ymgme.2026.109765)
Supplement: 2 [file NIHMS2164954-supplement-2.docx]

**Supplemental Table 1:** Selection of Clinical Outcome Assessments (COAs)- Physician Panel Focus Group Discussion (N= 5)

| **COAs** | **COIs Discussed** | **COI Achieving Consensus** |
| --- | --- | --- |
| AGS Severity Scale | Floor mobility Neurologic dysfunction Postural function (head and trunk) | Postural Function; Neurologic Function |
| Burke Fahn Marsden Dystonia Rating Scale | Neurologic dysfunction |  |
| Denver Developmental Scale -2nd edition | Complexity of verbal language | Complexity of verbal language |
| Eye tracker (Preferential Looking Protocols) | Preferential looking | Preferential looking |
| Hammersmith Infant Neurologic Examination (HINE) | Floor mobility Neurologic dysfunction Postural function (head and trunk) | Postural Function; Floor mobility; Neurologic Function |
| Patient-Reported Outcomes Measurement Information System (PROMIS) | Postural function (head and trunk) Fatiguability (Endurance) in completion of motor tasks |  |
| Wearables | Postural function (head and trunk) Fatiguability (Endurance) in completion of motor tasks Floor mobility | Floor mobility |
| WHO Developmental Milestones | Floor mobility Neurologic dysfunction Postural function (head and trunk) |  |
| **Legend:** Shaded green indicates failure to pass consensus, and Unshaded green indicates consensus achievement | | |
