# Supplementary material for: A novel patient-Centered approach to clinical trial readiness in rare diseases: Application in Aicardi-Goutières Syndrome (AGS)
Source: Mol Genet Metab. Author manuscript; Available in PMC 2026 May 12. (PMC13162174; doi:10.1016/j.ymgme.2026.109765)
Supplement: 3 [file NIHMS2164954-supplement-3.docx]

**Supplemental Table 2:** Selection of Clinical Outcome Assessments (COAs)- Neuropsychological Panel Focus Group Discussion (N= 5)

| **COAs** | **COIs Discussed** | **COI Achieving Consensus** |
| --- | --- | --- |
| Adaptive Behavior Assessment System -3rd edition (ABAS-3) | Independence in completion of activities of daily living |  |
| Bayley Scales of Infant and Toddler Development- 4th edition | Complexity of verbal language Postural function (head and trunk) Pointing with whole hand or finger Preferential looking Floor mobility Endurance and quality in completion of cognitive tasks | Complexity of verbal language; Postural Function; Floor mobility |
| Clinical Evaluation of Language Fundamentals- 5th edition (CELF-5) | Complexity of verbal language |  |
| Communication and Symbolic Behavior Scale (CSBS)- Caregiver section | Imitation of activities |  |
| Communication and Symbolic Behavior Scale (CSBS)- Performance section | Imitation of activities |  |
| Communication Matrix | Communication through behavior |  |
| Developmental Neuropsychological Assessment- 2nd edition (NEPSY-II) | Imitation of activities Endurance and quality in completion of cognitive tasks Fine motor speed | Fine motor speed |
| Leiter International Scales- 3rd edition | Neurologic dysfunction Use of adaptive/alternative communication Endurance and quality in completion of cognitive tasks |  |
| Preschool Language Scales -5th edition (PLS-5) | Complexity of verbal language |  |
| Vineland Adaptive Behavior Scale- 3rd edition | Complexity of verbal language Postural function (head and trunk) Communication through behavior Coordination in play Imitation of activities Independence in completion of activities of daily living Neurologic dysfunction Preferential looking | Communication through behavior; Complexity of verbal language; Imitation of activities; Independence in completion of activities of daily living |
| Delis-Kaplan Executive Function System, (D-KEFS) | Endurance and quality in completion of cognitive tasks |  |
| Fagan Test of Infant Intelligence (FTII) | Endurance and quality in completion of cognitive tasks Preferential looking |  |
| NIH Toolbox | Fatiguability (Endurance) in completion of motor tasks Endurance and quality in completion of cognitive tasks |  |
| Repeatable Battery for the Assessment of Neuropsychological Status | Endurance and quality in completion of cognitive tasks |  |
| Teller Acuity Cards | Preferential looking |  |
| Wide Range Assessment of Memory and Learning (WRAML) | Endurance and quality in completion of cognitive tasks |  |
| **Legend:** Shaded green indicates failure to pass consensus, and Unshaded green indicates consensus achievement | | |
