# Supplementary material for: A novel patient-Centered approach to clinical trial readiness in rare diseases: Application in Aicardi-Goutières Syndrome (AGS)
Source: Mol Genet Metab. Author manuscript; Available in PMC 2026 May 12. (PMC13162174; doi:10.1016/j.ymgme.2026.109765)
Supplement: 4 [file NIHMS2164954-supplement-4.docx]

**Supplemental Table 3:** Selection of Clinical Outcome Assessments (COAs)- Therapist Panel Focus Group Discussion (N= 6)

| **COAs** | **COIs Discussed** | **COI Achieving Consensus** |
| --- | --- | --- |
| 9-Hole peg | Coordination in play Fine motor speed | Fine motor speed |
| Adaptive Behavior Assessment System -3rd edition (ABAS-3) | Independence in completion of activities of daily living |  |
| Box and Blocks Shuttle (B&B) | Fatiguability (Endurance) in completion of motor tasks |  |
| Child Engagement in Daily Life | Coordination in Play |  |
| Child Pictorial Rating Scales | Fatiguability (Endurance) in completion of motor tasks |  |
| Functional Dexterity Test (FDT) | Fine motor speed |  |
| Goal-Oriented Assessment of Life skills (GOAL) | Independence in completion of activities of daily living |  |
| Movement Assessment Battery for Children- 2nd edition | Coordination in Play |  |
| Neonatal Assessment Visual European Grid (NAVEG) | Visual Function |  |
| Observer Reported Communication Ability (ORCA) | Communication through behavior Complexity of verbal language Use of adaptive/alternative communication |  |
| Peabody Developmental Motor Scales- 2nd edition | Coordination in Play Floor mobility Pointing with whole hand or finger Postural function (head and trunk) Visual function |  |
| Peabody Developmental Motor Scales- 3rd edition | Coordination in Play Floor mobility Pointing with whole hand or finger Postural function (head and trunk) Visual function" |  |
| Peabody Picture Vocabulary Test- 4th edition (PPTV-4) | Complexity of verbal language |  |
| Pediatric Evaluation of Disability Inventory Computer Adaptive Test (PEDICAT) | Independence in completion of activities of daily living | Independence in completion of activities of daily living |
| Screen-Q | Use of electronic devices |  |
| Test of Playfulness | Coordination in Play |  |
| Typing Assessment | Fatiguability (Endurance) in completion of motor tasks |  |
| Waisman Activities of Daily Living Scale (W-ADL) | Independence in completion of activities of daily living |  |
| 5X Sit-to-Stand test (5XSST) | Fatiguability (Endurance) in completion of motor tasks |  |
| 6 Minute Walk Test (6MWT) | Fatiguability (Endurance) in completion of motor tasks | Fatiguability (Endurance) in completion of motor tasks |
| Alberta Infant Motor Scale (AIMS) | Floor mobility |  |
| Gross Motor Function Measure-88 (GMFM-88) | Floor mobility Neurologic dysfunction Postural function (head and trunk) | Floor mobility, Postural function (Head and trunk) |
| 2 Minute Walk Test (2MWT) | Fatiguability (Endurance) in completion of motor tasks |  |
| **Legend:** Shaded green indicates failure to pass consensus, and Unshaded green indicates consensus achievement | | |
